# Trends in US Kidney Transplantation During the COVID-19 Pandemic

**DOI:** 10.7759/cureus.12075

**Published:** 2020-12-14

**Authors:** Stephen J Bordes, Lisandro Montorfano, Wesley West-Ortiz, Roberto Valera, Alejandro Cracco, Mileydis Alonso, Antonio D Pinna, Samer Ebaid

**Affiliations:** 1 Department of Anatomical Sciences, St. George's University School of Medicine, St. George's, GRD; 2 Transplant, Cleveland Clinic Florida, Weston, USA; 3 Transplant, American University of the Caribbean School of Medicine, Cupecoy, SXM

**Keywords:** covid-19, sars-cov-2, kidney transplantation, kidney transplant, living donor program, living donor kidney transplant, deceased donor kidney transplant, pandemic

## Abstract

Solid organ transplants have been impacted significantly during the COVID-19 pandemic in the United States. Limited data exist regarding changes in living donor kidney transplants. The aim of this study was to describe national trends in kidney transplantation during COVID-19. This descriptive cross-sectional study used publicly available data from the United Network for Organ Sharing (UNOS) and the National Kidney Registry (NKR). Plots of national waitlist inactivations, waitlist additions, deceased donor transplants and living donor transplants were created. An Auto Regressive Integrated Moving Average (ARIMA) model with interrupted time series analysis adjusting for first-order autocorrelation was used to evaluate for significant changes in outcome trends every four-week period during the COVID-19 era between March 15 and August 1, 2020. A statistical significance of 0.05 (𝛼) was established for analysis. Changes in kidney transplant volumes during the COVID-19 outbreak were registered. Density mapping and linear regression with interrupted time series analysis were used to characterize changes over time nationwide. Kidney transplants were affected significantly in recent months due to COVID-19. Deceased donor and living donor kidney transplant trends are described in this paper in addition to operative recommendations.

## Introduction

The novel 2019 coronavirus (SARS-CoV-2 or COVID-19) is considered the pandemic of the current century [1]. The virus rapidly spread worldwide and has been recognized as a global public health crisis due to a shortage of resources to care for the growing number of patients with acute respiratory distress syndrome (ARDS) and other complications resulting from SARS-CoV-2 [2,3]. By July 30, 2020, 136,577 deaths related to COVID-19 had been reported in the United States, posing another obstacle to an already challenging delivery of care with renal transplantation [4]. To potentially minimize virus exposure to both living organ donors and transplant recipients, programs temporarily postponed some or all living donor kidney transplants.

There are several ways to address end-stage renal disease (ESRD) depending on patient preference and severity of disease. Patients can receive lifelong hemodialysis or peritoneal dialysis, or they can be listed for kidney transplantation. Living donor nephrectomy was introduced to address the shortage of deceased donor kidneys [5]. The first deceased donor kidney transplant was accomplished in 1945, followed by the first living donor kidney transplant in 1953 and the first living-related renal transplant in 1954 [6,7]. The availability of organs remains the most significant factor limiting kidney transplants in ESRD patients.

COVID-19 has impacted transplantation centers globally. Many hospitals suspended transplant procedures to varying degrees due to concerns of infection transmission, resource allocation, and immunosuppression complications [8]. While much remains to be discovered regarding COVID-19, its early impact on transplantation is both profound and underrecognized. According to data released by the United Network for Organ Sharing (UNOS), 2019 marked the 7th record-setting year for transplants in the United States as can be recognized from an 8.7% increase in overall transplants, a 10.7% increase in deceased donor transplants, and a 5.8% increase in living donor transplants from 2018 [9]. While 2020 could once again mark another record-setting transplant year, temporary suspension of the living donor program in March proved to be a significant setback as living donations fell by nearly 50% overall for the 2020 year-to-date [9].

Over the last few months, a robust number of articles have been published describing the devastating impact of this virus in the world of surgery [8,10-14]. However, to the best of our knowledge, this is the first report describing the impact of COVID-19 on living donor kidney transplants and necessitates particular attention.

Although we recognize the morbidity and mortality rates of the novel coronavirus SARS-CoV-2 (COVID-19) pandemic, we bring to attention our concern regarding the shutdown of living donor kidney transplant programs. 23,401 kidney transplants were performed in 2019. Nevertheless, the waiting list remains substantially larger than the supply of donor kidneys [15]. Unfortunately, those who remain on the waiting list require renal replacement therapy for the treatment of ESRD. Even during non-pandemic times, patients on hemodialysis are 10 to 30 times more likely to expire compared to the general population [16,17]. With this in mind, our article aims to assess the changes in living donor kidney transplant volumes from the pre-COVID-19 (January 1, 2019 - March 14, 2020) to the present COVID-19 era (March 15 - August 1, 2020) and provide recommendations regarding safe living donor kidney transplantation during the COVID-19 pandemic while simultaneously avoiding future interruption of living donor programs throughout the nation.

## Materials and methods

Renal transplant data was publicly obtained from the United Network for Organ Sharing (UNOS) and the National Kidney Registry (NKR). Year-to-date living and deceased kidney donor transplants were charted and compared to trends from 2019. Data was broken down in terms of total 2019 renal transplants, total 2020 year-to-date transplants per week, 2020 living donor year-to-date transplants per week, and total 2020 deceased donor year-to-date transplants per week. Year-to-date waitlist additions and inactivations were also considered. Given that data were publicly available with deidentified information, institutional review board (IRB) approval and informed consent were not required as per institutional policy.

On March 11, 2020 the Novel Coronavirus Disease (COVID-19), was declared a pandemic by the World Health Organization (WHO). On March 13, 2020, a national emergency was declared in the United States concerning the COVID-19 Outbreak. As of March 15, 2020, UNet (the electronic system managed by UNOS that allows transplant professionals to submit, store, and manage transplant-associated data) users could denote if waitlist inactivations were due to COVID-19 precautions. Therefore, for this analysis, the period from January 1, 2019 to March 14, 2020 was designated the pre-COVID-19 era while the period from March 15 to August 1, 2020, was designated as the present COVID-19 era.

International Business Machines (IBM) Statistical Product and Service Solutions (SPSS) software (Armonk, NY) was used for statistical analysis. Plots of national waitlist inactivations, waitlist additions, deceased donor transplants and living donor transplants were created using spreadsheets. An Auto Regressive Integrated Moving Average (ARIMA) model with interrupted time series analysis adjusting for first-order autocorrelation was used to evaluate for significant changes in outcome trends every 4-week period during the COVID-19 era. A statistical significance of 0.05 (𝛼) was established for analysis.

## Results

Changes in 2020 monthly data

There were a total of 5,071 transplants in the 2020 pre-COVID-19 era compared to 7,799 during the pandemic, which represented an increase of 53.8%, a lower increase compared to the 97.6% increase found in the 2019 data for the same periods. For the 2020 year to year data, we found a trend of 958.54 new transplants per month (p < 0.001), higher than the 901.01 transplants per month trend of the last year (p ˂ 0.001).

**Figure 1:**
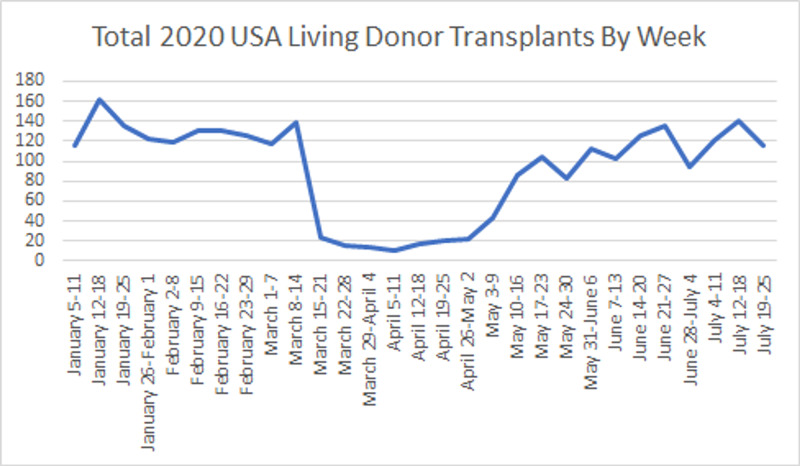
Total 2020 living donor kidney transplants in the United States by week.

**Figure 2:**
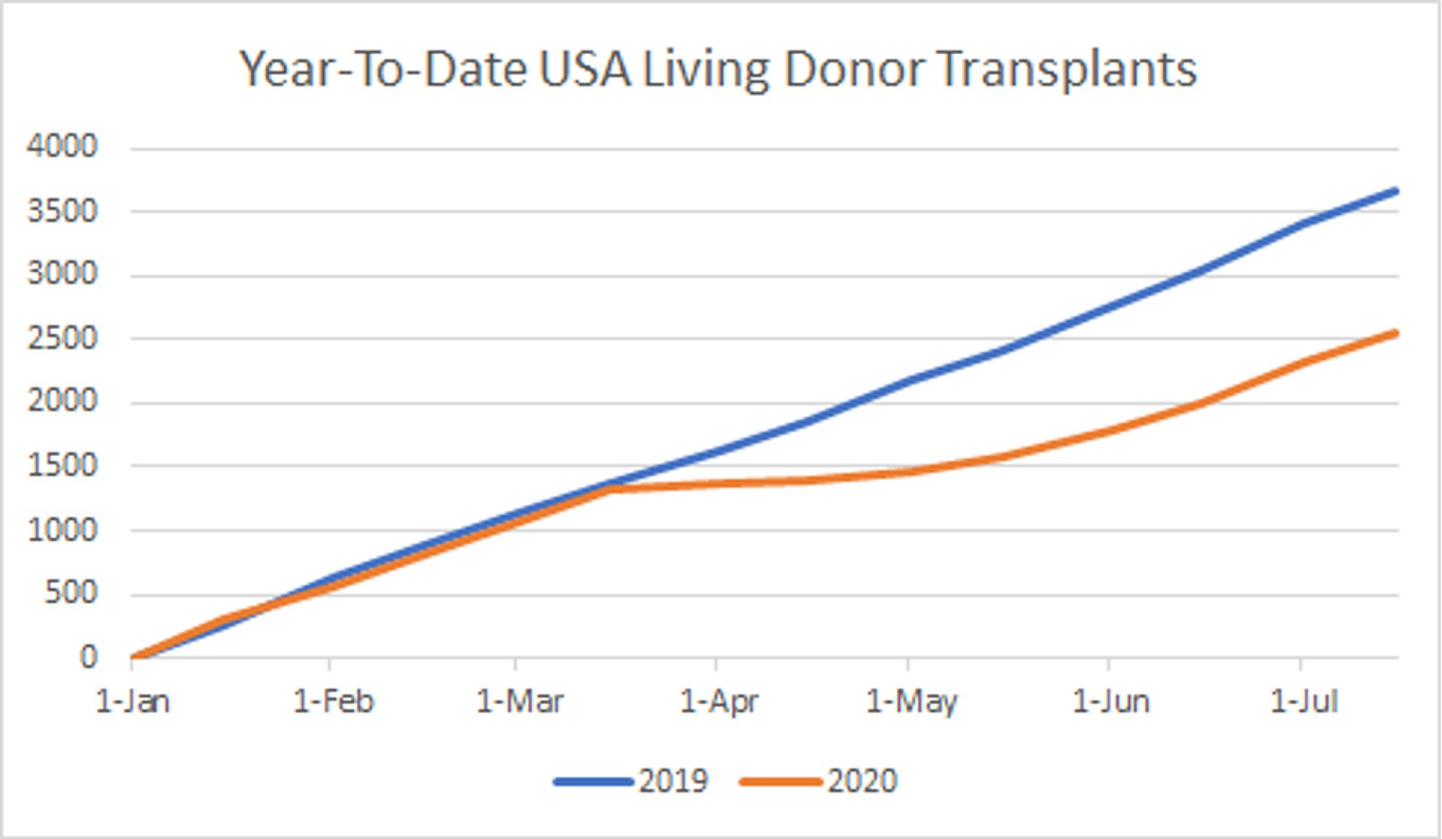
Total year-to-date living donor kidney transplants in the United States.

There were a total of 1,329 living donor transplants in 2020 pre-COVID-19 compared to 1508 during the pandemic, which represented an increase of 13.4%, compared to the 43% increase found in the 2019 data for the same periods. For the 2020 data, we found a trend of 195.13 new transplants per month (p ˂ 0.001), lower than the 286.77 transplants per month trend of the last year (p ˂ 0.001) (Tables 1, 2; Figures 1, 2).

**Table 1:** Trends in year-to-date total, deceased donor, and living donor transplants in 2019. Δ (delta), difference in value.

	January 5 - March 15	March 15 – August 1	Δ%	Estimate of effect	Standard Error	p-value
Total	4527	8945	+97.6	+958.54	14.83	˂0.001
Deceased	3149	6228	+97.8	+670.63	4.94	˂0.001
Living	1378	2717	+97.2	+286.77	9.73	˂0.001

**Table 2:** Trends in year-to-date total, deceased donor, and living donor transplants in 2020. Δ (delta), difference in value.

	Pre COVID-19	COVID-19	Δ%	Estimate of effect	Standard Error	p-value
Total	5071	7799	+53.8	+901.01	34.45	˂0.001
Deceased	3742	6291	+68.1	+704.29	20.38	˂0.001
Living	1329	1508	+13.4	+195.13	16.49	˂0.001

**Figure 3:**
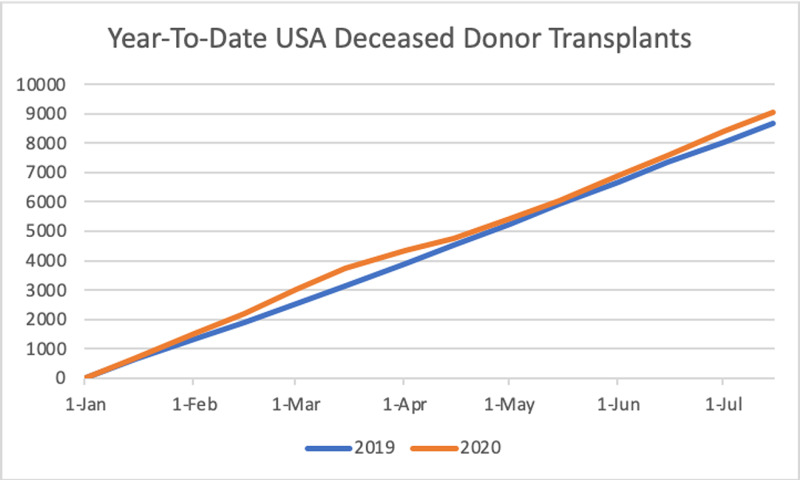
Total year-to-date deceased donor kidney transplants in the United States.

**Figure 4:**
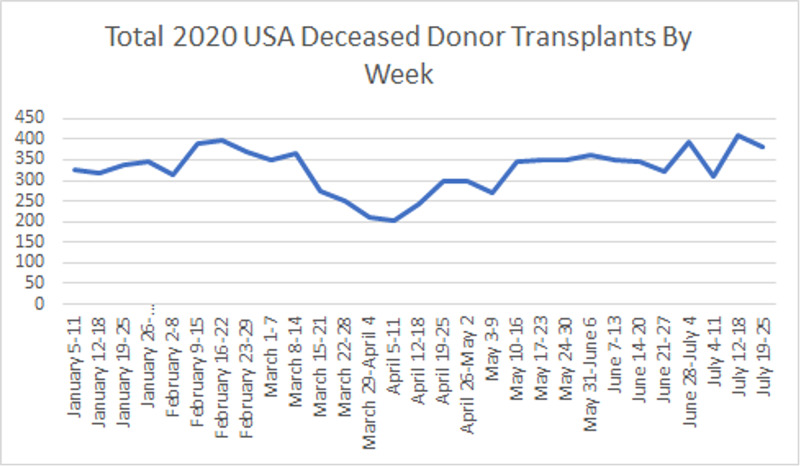
Year-to-date deceased donor kidney transplants in the United States by week.

Lastly, the total number of deceased donor transplants pre-COVID-19 era was 3742 compared to 6,291 during the pandemic. This represented an increase of 68.1%, a lower increase compared to the 97.8% increase found in the 2019 data for the same periods. However, we found a trend of 704.29 new transplants per month (p < 0.001), higher than the 670.63 transplants per month trend of the last year (p < 0.001) (Figures 3, 4).

Changes in 2020 weekly data

We found significant changes in the deceased donor trends for the first three 4-week periods of the COVID-19 era. The mean difference in deceased donor transplants per week (2019 vs. 2020) for the periods March 15 - April 11, April 12 - May 10, and May 11 - June 7 were -148.82 (CI = -206.29; -91.34, p ˂ 0.001), -137.34 (CI = -217.58; -57.10, p ˂ 0.001) and -125.87 (CI = -234.50; -17.24, p=0.022) (Table 3).

**Table 3:** Changes in trend of deceased donor transplants per four-week period during the COVID-19 era.

Period	Estimate of effect	Standard Error	Confidence Interval	p-value
Pre-COVID-19 era	+5.91	3.70	-1.87; 13.70	0.122
March 15 – April 11	-148.82	27.24	-206.29; -91.34	˂0.001
April 12 – May 10	-137.34	38.03	-217.58; -57.10	0.001
May 11 – June 7	-125.87	51.49	-234.50; -17.24	0.022
June 8 – July 4	-114.40	66.00	-253.65; 24.85	0.096
July 4 – July 25*	-102.94	81.01	-273.84; 67.97	0.216

Regarding living donor transplants, the first two 4-week periods of the COVID-19 era showed a significant decrease. Between March 15 and April 11, a mean decrease of 120.37 cases per week was found (CI = -152.58; -88.16, p ˂ 0.001). The April 12 - May 10 period showed a decrease of 87.99 cases per week (CI = -133.27; -42.71, p ˂ 0.001) (Table 4).

**Table 4:** Changes in trend of living donor transplants per four-week period during the COVID-19 era.

Period	Estimate of effect	Standard Error	Confidence Interval	p-value
Pre-COVID-19 era	-0.71	2.10	-5.10; 3.68	0.737
March 15 – April 11	-120.37	15.33	-152.58; -88.16	˂0.001
April 12 – May 10	-87.99	21.55	-133.27; -42.71	˂0.001
May 11 – June 7	-55.61	29.18	-116.90; 5.69	0.068
June 8 – July 4	-23.26	37.35	-101.69; 55.24	0.539
July 4 – August 1	+9.16	45.78	-87.02; 105.33	0.843

**Figure 5:**
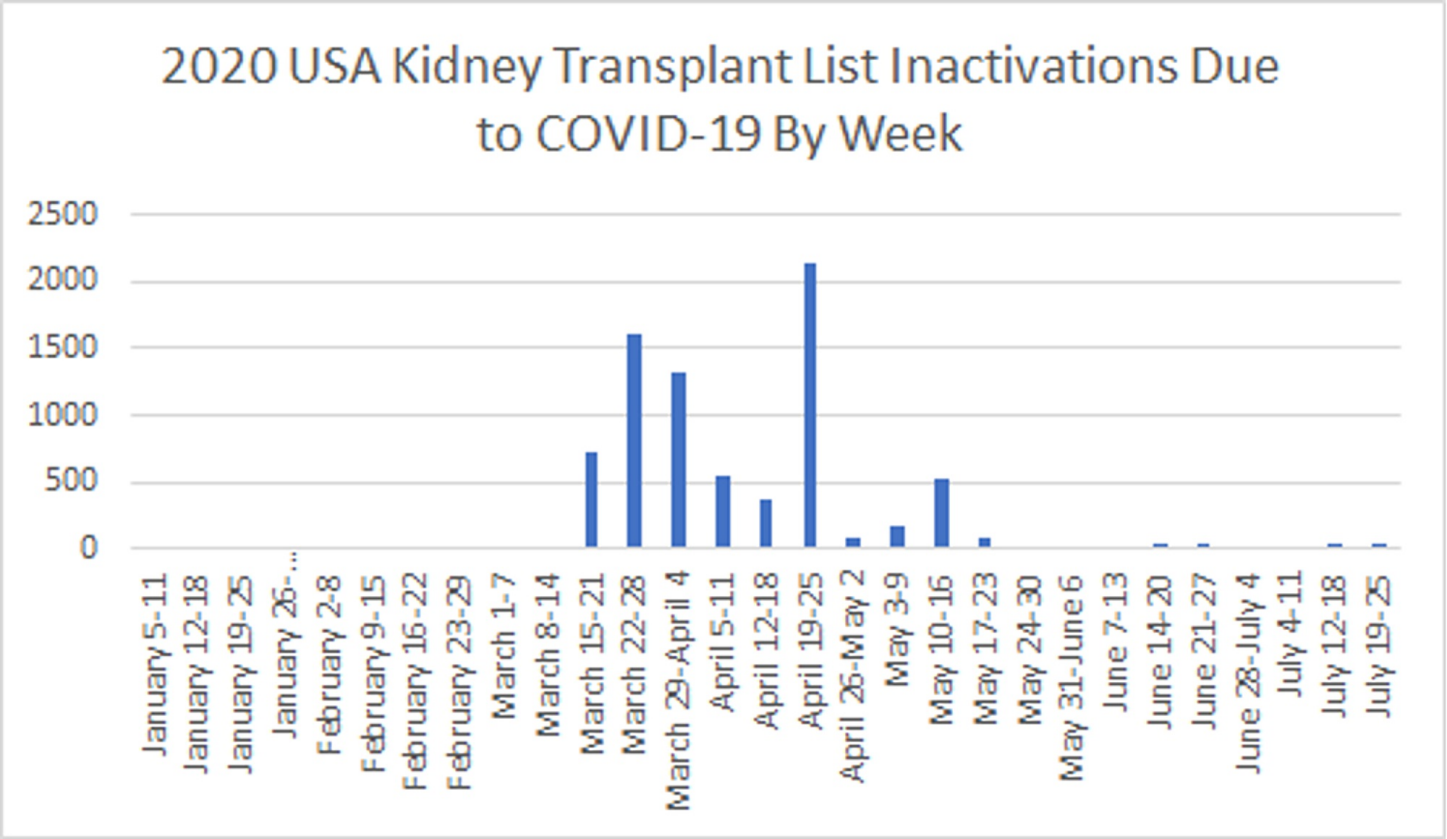
Year-to-date kidney transplant inactivations due to COVID-19 in the United States by week.

**Figure 6:**
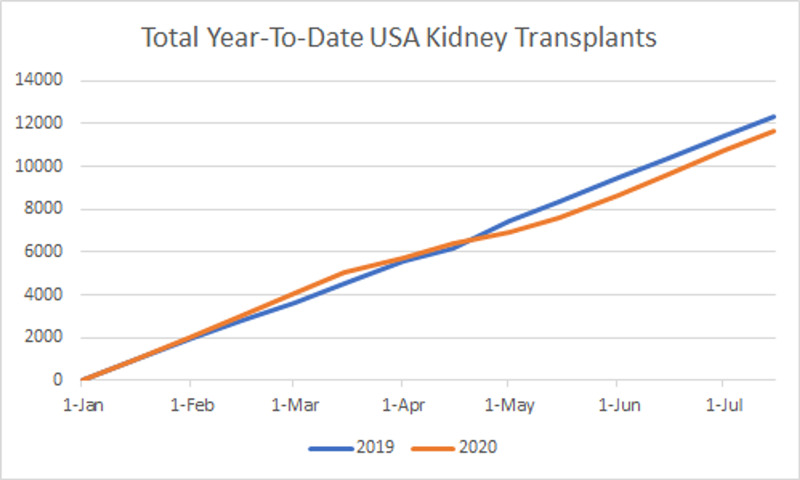
Total year-to-date kidney transplants in the United States.

Finally, we analyzed the changes in the trends of waitlist inactivations up to August 1. A total of 19,431 inactivations were reported during the COVID-19 era, of which 7,846 (40.38%) were due to SARS-CoV-2 infection. We found that the number of all-cause inactivation per week increased for the March 15 - April 11 and April 12 - May 10 periods, with 1596.07 (CI = 901.23; 2,290.94, p ˂ 0.001) and 1,333.12 (CI = 356.24; 2,310.01, p = 0.008) inactivations per week respectively. The trend of COVID-19-related inactivations change significantly only during the March 15 - April 11 period (+1080.42 inactivations per week, CI = 493.92; 1,666.93, p = 0.001) (Tables 5, 6; Figures 5, 6).

**Table 5:** Changes in trend of all-cause inactivations per four-week period during the COVID-19 era.

Period	Estimate of effect	Standard Error	Confidence Interval	p-value
March 15 – April 11	+1596.07	330.74	901.23; 2290.94	˂0.001
April 12 – May 10	+1333.12	464.98	356.24; 2310.01	0.008
May 11 – June 7	+1070.16	629.39	-252.13; 2392.45	0.101
June 8 – July 4	+807.20	805.70	-885.52; 2499.92	0.326
July 4 – August 1	+544.24	987.57	-1530.57; 2619.04	0.586

**Table 6:** Changes in trend of COVID-19-related inactivations per four-week period during the COVID-19 era.

Period	Estimate of effect	Standard Error	Confidence Interval	p-value
March 15 – April 11	+1080.42	279.17	493.92; 1666.93	0.001
April 12 – May 10	+808.75	392.74	-16.37; 1633.90	0.050
May 11 – June 7	+537.05	530.47	-577.42; 1651.51	0.321
June 8 – July 4	+266.01	677.75	-1157.88; 1689.90	0.698
July 4 – August 1	-5.577	829.536	-1748.37; 1737.21	0.995

## Discussion

Herein, we describe national trends in kidney transplants during the COVID-19 pandemic. COVID-19 has been negatively impacting solid organ transplantation services worldwide. As the pandemic continues to progress, transplant centers face numerous challenges that have led to a steep decline in all solid organ transplants. As of August 1, 2020, the United States has registered 22,208 total solid organ transplants. From January 2019 to December 2019, the United States recorded 39,719 total transplants, an 8.7% increase from 2018 which recorded 36,269 total solid organ transplants [18]. Currently, deceased donor kidney year-to-date transplants are outpacing those from the same period in 2019 (9,982 between January 1, 2020 and August 1, 2020 vs. 9,329 between January 1, 2019 and August 1, 2019; Figure 3). However, deceased donor kidney transplantations decreased by 35% in April due to COVID-19 procurement measures [19]. By June 2, 2020, deceased donor kidney transplants returned to 95% of pre-COVID era numbers [20]. The kidney living donor program came to a near halt between March 15 and May 9, 2020. The National Kidney Registry reported an 89% drop in living donor transplants in April, which was due to concerns surrounding laparoscopy and donor safety during the COVID-19 pandemic [19]. Living donor transplants returned to 71% of pre-COVID-19 rates as of June 2, 2020 and kidney paired donations reached levels of 63%. A surge in kidney transplants is predicted in the Fall of 2020 due to a delay in kidney paired donations [20].

A study on COVID-19 transmission dynamics in Wuhan, China reports that human-to-human transmission among close contacts is a primary method of virus spread [21]. Due to potential droplet transmission by asymptomatic patients, COVID-19 can be spread rapidly within the healthcare community. Various COVID-19 outbreaks have been reported in multiple hospital settings [22-27]. A report from Wuhan showed 12% of 138 inpatient COVID-19 cases were acquired during hospital admissions [28]. Other hospitals reported similar numbers [22,28]. Living donor kidney transplant procedures may allow 4 to 5 hours of continuous COVID-19 aerosol exposure. We believe the potential risk of transmission due to aerosolization of the virus while performing laparoscopic surgery and the risk of viral transmission from healthcare workers and COVID-19 positive patients to living donor transplant donors and recipients were the main concerns that drove transplant centers to perform fewer living donor kidney transplants during the COVID-19 outbreak.

Based on our review of the available data and new transplant guidelines, we strongly recommend that every hospital in which COVID‐19 patients are treated and living donor transplants are performed should ensure that patients have at least two negative COVID-19 tests prior to surgery (14 days and 24-72 hours pre-operatively) in addition to a period of self-quarantine leading up to organ donation [29]. At our institution, we routinely perform three COVID-19 respiratory nucleic amplification tests (NATs), including a third rapid COVID-19 test on the morning of surgery.

Proper donning and doffing in the anteroom using adequate personal protective equipment should be employed by physicians that perform surgeries on all patients, regardless of COVID-19 status. This includes N95 mask, cap, boot covers, sterile gowns, gloves, face and eye protection covers. In order to minimize the risk of viral transmission between COVID-19 positive patients, transplant patients, and hospital staff, a minimum number of staff should be present for the duration of COVID-19 related procedures. This includes one lead surgeon and one to two operating assistants. We recommend one circulating nurse, one anesthetist, and one scrub technician.

Another measure that can be undertaken in order to minimize the risk of viral transmission studies is performing laparoscopic surgery in negative-pressure operating rooms with an in-room high-efficiency particulate air (HEPA) filter to ensure maximum air purification as recommended in previous studies [11]. Generally, viruses travel inside air droplets that comprise the aerosol. Nearly all aerosols are removed by modern ventilation HEPA systems that filter operating rooms [11]. Prior studies at our institution also highlight precautions for toxic aerosolized pneumoperitoneum during laparoscopy. These additional recommendations include minimal abdominal carbon dioxide (CO2) insufflation, minimal use of trocars (2 recommended), desufflation of toxic aerosols, and plastic draping of the patient with underlying HEPA filtration [11].

The average waitlist time for a kidney transplant is three to five years and depends on a variety of factors. Patients with a high panel reactive antibody (PRA) are even less likely to find an organ match due to higher immunoreactivity and thus higher chances of organ rejection. Some transplant centers across the country have reported cases of patients with high PRA that have lost their opportunity to receive a kidney transplant due to the fact that they tested positive for COVID-19 the day of the surgery. Unfortunately, those who remain on the waiting list require renal replacement therapy for treatment of ESRD. For the majority of patients with end-stage renal disease, hemodialysis is the mainstay of treatment. Three times per week, hemodialysis patients remain indoors for 4 hours in frequent contact with other patients and healthcare staff increasing their risk of acquiring COVID-19. Morbidity and mortality rates in dialysis patients are significantly higher compared to the non-dialysis population. In April 2020, the kidney transplant waitlist patient mortality rate increased by 43.0% based on available UNOS data. A 170%, 89%, and 54% solid organ transplant waitlist all-cause mortality increase was reported in UNOS regions 9, 1, and 2, respectively [30]. Northeast Region 2, which includes New York, noted a two-fold increase in kidney waitlist mortality [30]. For these reasons, we recommend no future suspension of living donor transplants due to reasons attributed to COVID-19 if the above safety procedures are employed. Risks to both patients and staff remain acceptable when adequate precautions are undertaken.

## Conclusions

COVID-19 has had direct and indirect associations with kidney transplantation in the United States. This article described national trends in kidney transplants during the COVID-19 pandemic. While the overall kidney transplant rate has remained stable year-to-date, living donor transplants decreased drastically at the onset of the pandemic. Although there is no single, universally agreed upon set of answers, we have presented what we believe is a balanced, compelling description of safe approaches to operate on COVID-19 patients and prevent living donor kidney transplantation contagions during the current COVID‐19 outbreak. Transplant surgeons should be prepared for COVID-19 related kidney transplant inactivations and waitlist mortalities related to ESRD. In summary, we recommend no future suspension of the living donor program and no future transplant interruptions for high PRA recipients as chances of secondary organ match are low.
